# Utility of high-resolution magnetic resonance vessel wall imaging in differentiating between atherosclerotic plaques, vasculitis, and arterial dissection

**DOI:** 10.1007/s00234-022-03093-7

**Published:** 2022-12-02

**Authors:** Oranan Tritanon, Suphanika Mataeng, Mungkorn Apirakkan, Theeraphol Panyaping

**Affiliations:** 1 Division of Diagnostic Neuroradiology, Department of Diagnostic and Therapeutic Radiology, Faculty of Medicine, Ramathibodi Hospital, Mahidol University, 270 Rama VI Road, Thung Phaya Thai, Ratchathewi, Bangkok, 10400 Thailand; 2 Department of Diagnostic and Therapeutic Radiology, Faculty of Medicine, Ramathibodi Hospital, Mahidol University, 270 Rama VI Road, Ratchathewi, Bangkok, 10400 Thailand

**Keywords:** Atherosclerosis, Vasculitis, Dissection, Vessel wall imaging

## Abstract

**Purpose:**

Differentiating between atherosclerosis, vasculitis, and dissection is a diagnostic challenge because of inconclusive findings on conventional imaging and some overlap in the vessel wall patterns. The aim of this study was to determine whether vessel wall MRI patterns can differentiate between these vasculopathies.

**Methods:**

We retrospectively reviewed 3T high-resolution vessel wall imaging studies of patients diagnosed with atherosclerotic plaques, vasculitis, and dissection. The patterns of involvement, wall enhancement, and T1 and T2 signals, as well as the specific patterns, were assessed and compared between the three diseases.

**Results:**

Fifty-nine patients with atherosclerosis (*n* = 33), vasculitis (*n* = 13), and dissection (*n* = 13) were enrolled. There were significant differences in the pattern of involvement between the three groups (*P* < 0.001), with concentric wall thickening in vasculitis patients (84.6%) and eccentric wall thickening in atherosclerosis (97%) and dissection (92.3%) patients. There was also a significant difference in the specific pattern (*P* < 0.001), with intimal flap (76.9%) and intramural hematoma (23.1%) in dissection patients and intraplaque hemorrhage (18.2%) in atherosclerosis patients. Furthermore, subgroup analysis showed a significant difference in the wall enhancement pattern between atherosclerosis and vasculitis patients (*P* < 0.05). Finally, there was a significant difference in the location of involvement between the three groups (*P* < 0.001).

**Conclusion:**

By using the pattern of involvement, wall enhancement, and specific patterns, vessel wall MRI can help differentiate between atherosclerosis, vasculitis, and dissection.

## Introduction

Cerebrovascular disease is a leading international health problem. Approximately 80% of all cerebrovascular events are associated with ischemic stroke. About 8–50% of ischemic stroke is caused by atherosclerotic disease, the other causes of which include arterial dissection and intracranial vasculitis of several etiologies [1, 2].

Correct diagnosis of these vasculopathies is based on clinical data, laboratory investigation, and imaging. Traditional imaging methods for visualizing the intracranial and cervical arteries include intra-arterial digital subtraction angiography (DSA), computed tomographic (CT) angiography, Doppler ultrasonography (DUS), and MR angiography (MRA). However, most techniques could only depict the arterial lumen but do not adequately assess the vessel wall pathology and can be of limited value in differentiating the causes of intracranial vasculopathies.

In 2017, the principles and expert consensus recommendations from the American Society of Neuroradiology suggested use of vessel wall imaging (VWI) for differentiating between the causes of intracranial arterial narrowing, including intracranial atherosclerotic plaques, vasculitis, and arterial dissection [3]. The distinct pathophysiology of each disease provides different image patterns, with numerous studies reporting the benefits of VWI.

Vessel wall MRI of intracranial atherosclerotic plaque typically shows arterial wall thickening, with eccentric involvement of the arterial wall. The plaque adjacent to the lumen often shows hyperintense on T2-weighted images and may be enhanced [3–7]. In CNS vasculitis, vessel wall MRI often reveals smooth, homogeneous, concentric wall thickening, and enhancement [8–11]. However, vasculitis sometimes demonstrates eccentric wall abnormality [9]. Vessel wall MRI of intracranial arterial dissection shows a curvilinear hyperintensity on T2-weighted images separating the true lumen from the false lumen, representing intimal flap and eccentric arterial wall thickening with signal characteristics of blood, which represents intramural hematoma [3, 12–14].

Several previous studies have demonstrated the benefits of vessel wall imaging. Mossa-Basha et al. suggested that VWI can significantly improve the differentiation of non-occlusive intracranial vasculopathies when combined with traditional luminal imaging modalities [11]. They also reported in another study that intracranial atherosclerotic disease (ICAD) lesions probably had more eccentric wall involvement than vasculitic lesions. Combining T1 and T2 VWI could significantly increase the sensitivity of VWI in differentiating intracranial atherosclerotic disease from other vasculopathies from 90.1 to 96.3% [8]. However, the VWI ability to differentiate between atherosclerosis, vasculitis, and arterial dissection remains unclear.

To our knowledge, none of the previous literature had demonstrated the vessel wall’s ability to differentiate between intracranial and intracervical atherosclerosis, vasculitis, and arterial dissection. Herein, we examined whether VWI can differentiate between atherosclerosis, vasculitis, and dissection using the vessel wall patterns. We suggest that VWI is a non-invasive tool allowing precise diagnosis of these vasculopathies.

## Material and methods

### Patient population

Our local ethics committee approved this study. We retrospectively reviewed all patients diagnosed with atherosclerotic plaque, vasculitis, and dissection who underwent MRI/MRA of the brain/neck with VWI between January 2015 and December 2019 at Ramathibodi Hospital.

Medical records were reviewed for demographic data, clinical symptoms, and laboratory investigation. The following novel classification methods which based on other methods [8, 15–18] were required for final diagnosis. For atherosclerosis, patients were required to have (1) ≥ 2 vascular risk factors (e.g., hypertension, dyslipidemia, diabetic mellitus, and coronary artery disease), with addition of [2] atherosclerotic plaque confirmation on color Doppler ultrasonography or calcified plaque on CT and [3] no evidence of CNS infection/inflammation or short-term reversibility of arterial lesions, i.e., vasospasm or RCVS [8, 15]. For CNS vasculitis, patients were required to have [1] histological confirmation of vasculitis or [2] CSF evidence of infection/inflammation in combination with arterial stenosis and a serology test compatible with vasculitis or [3] classification/diagnostic criteria according to the American College of Rheumatology for specific type of vasculitis and [4] a treatment response after steroid/immunosuppressive drug administration [8, 16]. For arterial dissection, patients were required to have [1] conventional angiography or definite imaging findings on CTA and/or color Doppler ultrasonography for arterial dissection and [2] chronological changes on follow-up imaging [17, 18].

Inclusion criteria included patients who were diagnosed with atherosclerosis, arterial dissection, or CNS vasculitis with at least one intra-or extracranial arterial stenosis identifiable by DUS, CT/CTA, or angiography who also underwent 3D-TOF, CE-MRA sequences with VWI to evaluate cause of arterial stenosis, stroke in hidden area/posterior fossa, or other brain parenchymal lesions.

**Fig. 1:**
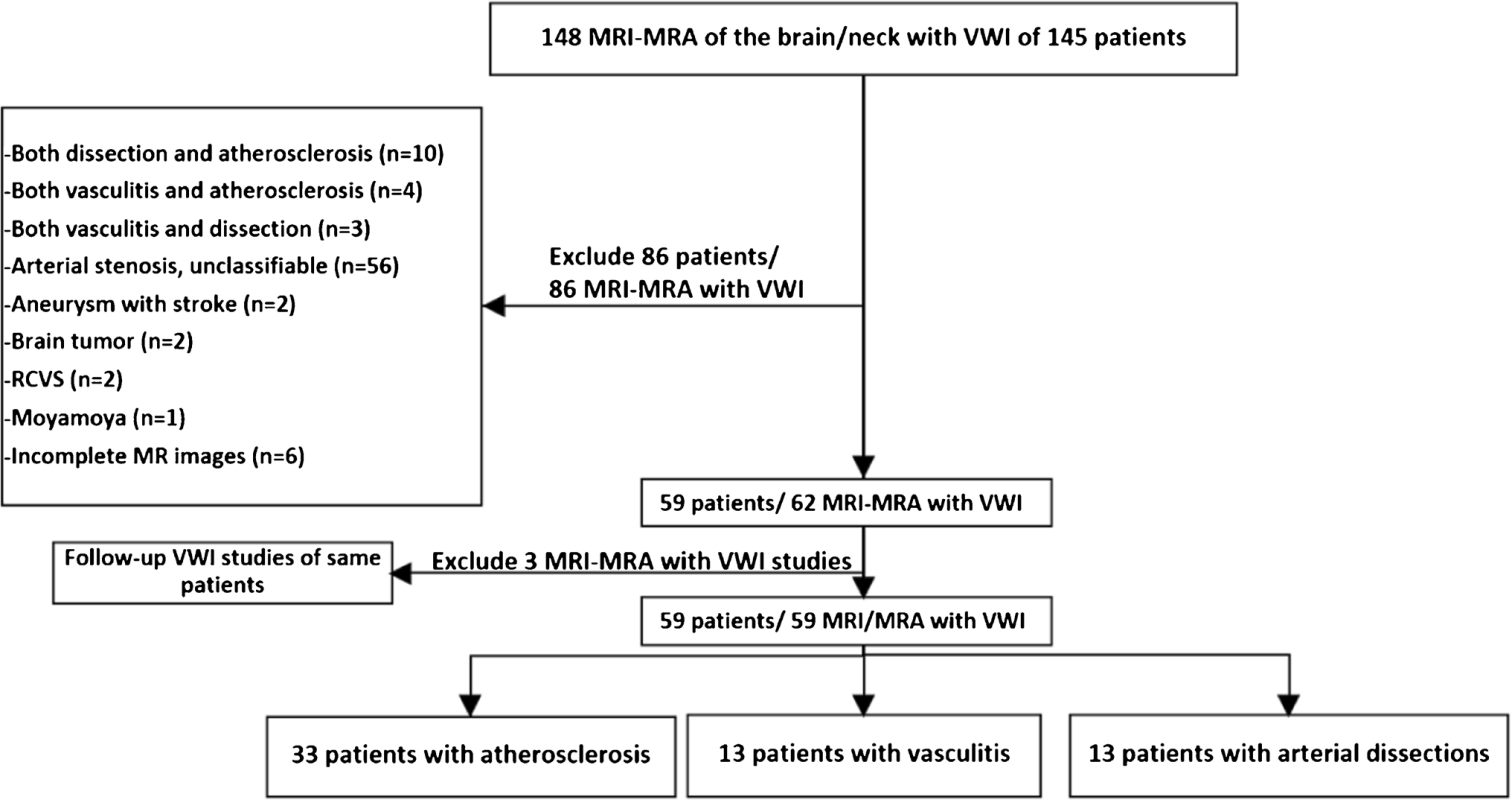
Flow chart of patient selection process

There were a total of 148 MRI/MRA of the brain/neck with VWI studies of 145 patients performed. Eighty-six patients were excluded. Of 86 excluded patients, 10 had both dissection and atherosclerosis, 4 had both vasculitis and atherosclerosis, 3 had both vasculitis and dissection, 56 had arterial stenosis not fulfilled aforementioned classification criteria, 2 had aneurysm with stroke, 2 had brain tumor, 2 had RCVS, 1 had Moyamoya, and 6 had incomplete MR images (Fig. 1). The patients were classified into each disease group by the second-year in-training neuroradiology fellow and a neurologist after reviewing medical records and laboratory investigations with blinded VWI.

A total of 59 patients were included; 33 were diagnosed with atherosclerosis, 13 were vasculitis, and 13 had arterial dissections. All patients were treated with experienced neurologists, and follow-up MRI/MRA studies were performed after 1–24 months depending on disease groups.

### Imaging protocol

MRI acquisition was performed on a 3T system (Ingenia 3T; Philips Healthcare, Best, the Netherlands) with a standard head coil. The MRI VWI protocol included pre-contrast and contrast-enhanced (CE) three-dimensional (3D) T1-weighted volumetric isotropic turbo spin-echo acquisition (repetition time/echo time, 350/18 ms; flip angle, 80°; field of view, 200 × 200 × 100 mm; acquisition spatial resolution, 1.0 × 1.0 × 1.0 mm^3^; reconstructed spatial resolution, 0.39 × 0.39 × 0.5 mm^3^; acquisition time, 6.25 min). Pre-contrast and CE-3D BrainVIEW black blood motion-sensitized driven equilibrium was also performed (repetition time/echo time, 700/35 ms; flip angle, 80°; field of view 200 × 251 × 183 mm; acquisition spatial resolution, 0.8 × 0.8 × 0.8 mm^3^; reconstructed spatial resolution, 0.58 × 0.58 × 0.4 mm^3^; acquisition time, 5.14 min). An additional T2-weighted VWI sequence was obtained in some patients. MRA was performed with 3D-TOF and CE-MRA techniques for luminal imaging.

### Image analysis

**Fig. 2:**
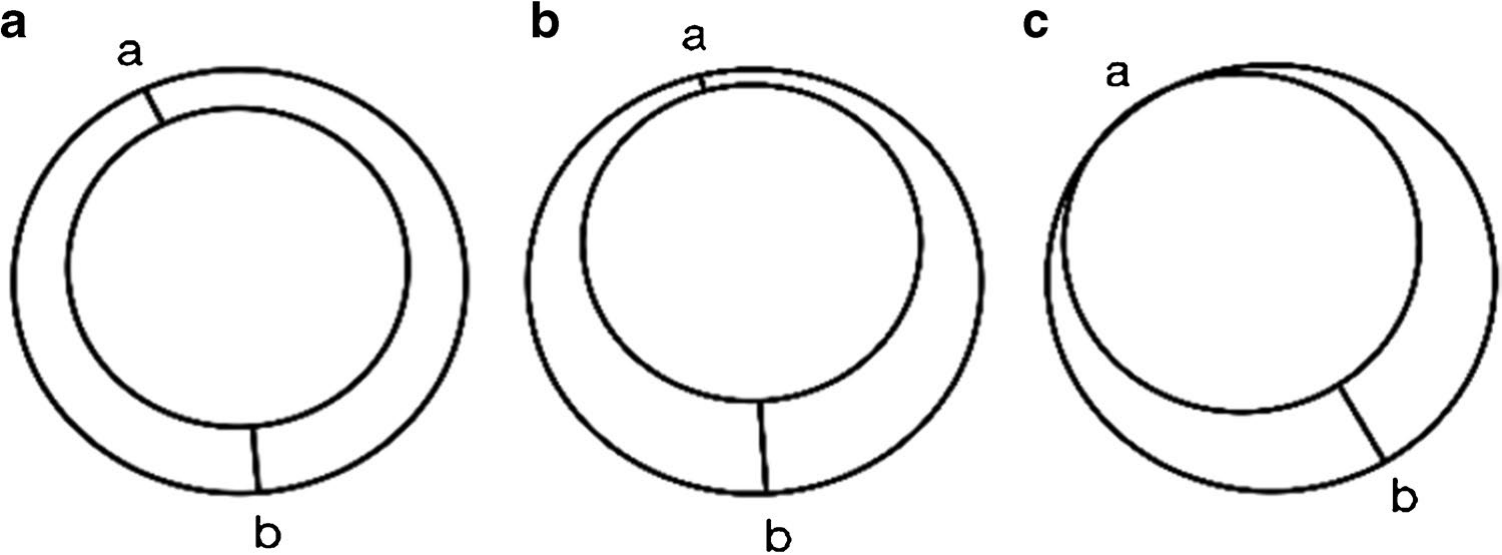
(a) Concentric lesion: a/b > 50% when “a” is the thinnest wall segment width and “b” is the thickest wall segment width. (b and c) Eccentric lesion: a/b < 50% when “a” is the thinnest wall segment width and “b” is the thickest wall segment width.

Two neuroradiologists blinded to the final clinical diagnosis independently evaluated whether the location of involvement was diffuse (> 3 vessels involved) or focal (≤ 3 vessels involved) and determined the site of most stenosis on MRA images. The radiologists then analyzed the VWI of each patient at the most stenotic point using multiplanar reconstruction. The pattern of wall involvement (eccentric or concentric), enhancement pattern (focal, heterogeneous, diffuse, or absent), and the specific vessel wall findings were documented. On imaging, concentric lesions were defined as those with the thinnest wall segment width > 50% of the thickest segment, while eccentric lesions were defined as those with the thinnest segment width < 50% of the thickest segment (Fig. 2).

For the enhancement pattern, “focal” was defined as the lesion with an area of enhancement < 50% of the entire thickened wall, “heterogeneous” was the lesion with scattered areas of enhancement of the thickened wall, “diffuse” was the lesion with an area of enhancement > 50% of the entire thickened wall, and “absent” was defined as no enhancement. Specific vessel wall findings were also included. Intramural hematoma was defined as a hyperintense T1W signal in the vessel wall. The intimal flap was defined as a curvilinear hyperintensity on T2W or post-contrast image separating the true and false lumen. The fibrous cap was a juxtaluminal band with T2 hyperintense or post-gadolinium enhancement. Intraplaque hemorrhage was defined as intraplaque T1 hyperintensity.

Any discrepancies between the two reviewers were solved by consensus in a separate reading session.

The degree of luminal stenosis of the most stenosed vessel in each case was then measured twice, according to the WASID criteria for intracranial arteries and the NASCET criteria for cervical arteries [19, 20].

### Statistical analysis

Clinical characteristics of each subject were summarized and compared between the three clinical diagnoses using the chi-square test and ANOVA. Vessel wall pattern assessment of the lesions was summarized and compared between the three clinical diagnoses using the chi-square test. Sensitivity and specificity were used to evaluate how well the imaging findings discriminated between vasculopathies. Kappa statistic (*κ*) was used to assess the intraobserver and interobserver agreement for the imaging findings. All statistical analyses were performed with statistical software (SPSS v26).

## Results

A total of 59 patients were enrolled in the present study (mean age ± SD, 58.2 ± 18.2 years; age range, 15–87 years; 31 men [52.5%], 28 women [47.5%]). Demographic data of patients was shown in Table 1.

**Table 1:** Demographic, laboratory, and imaging findings of patients

Code no	Age	Gender	Risk factors and underlying disease	Laboratory and imaging findings	Clinical presentation	Disease
1	62	F	HT, DLP		Right hemiparesis	Atherosclerosis
2	48	M	DLP		Headache	Dissection
3	58	F	HT, DM		Left hemiparesis	Atherosclerosis
4	73	M	HT, DM		Right paresthesia	Atherosclerosis
5	86	M	HT, DM		Transient visual loss	Atherosclerosis
6	63	M	HT, CAD		Left hemiparesis	Atherosclerosis
7	29	F	DLP	-ESR 62, CRP 17.21 -CTA: severe stenosis of left CCA and left ICA	Right hemiparesis blurred vision, left carotid bruit	Vasculitis (Takayasu arteritis)
8	72	M	HT, DLP		Right hemiparesis	Atherosclerosis
9	40	M	DLP		Left-sided ataxia	Dissection
10	43	F		-ESR 100 -Positive nRNP/Sm, positive anti-DNA -CSF: lymphocytic pleocytosis, elevated protein, normal glucose level	Dysarthria and left hemiballismus	Vasculitis (SLE with CNS vasculitis)
11	37	F	HT, DM, DLP		Right hemiparesis	Atherosclerosis
12	40	F	DLP	-ESR 131 -Positive anti-HIV -CSF: lymphocytic pleocytosis, elevated protein, normal glucose level	Left hemiparesis	Vasculitis (HIV with CNS vasculitis)
13	20	F		-ESR 51, CRP 10.16 -Positive nRNP/Sm, positive anti-DNA -CSF: lymphocytic pleocytosis, elevated protein, low glucose level	Progressive headache and night-awakening pain	Vasculitis (SLE with CNS vasculitis)
14	70	M	HT, DLP		Left hemiparesis	Atherosclerosis
15	79	M	HT, DM, DLP		Drowsiness	Atherosclerosis
16	87	M	HT, DM, CAD, renal tumor		Right arm weakness	Atherosclerosis
17	43	M	DLP	-ESR 50 -Positive anti HIV -CSF: lymphocytic pleocytosis, elevated protein, low glucose level	Right hemiparesis	Vasculitis (HIV with CNS vasculitis)
18	77	M	DM, DLP		Right hemiparesis	Atherosclerosis
19	44	F		-ESR 72 - Positive nRNP/Sm, positive anti-DNA -CSF: lymphocytic pleocytosis, elevated protein, low glucose level	Right facial paresthesia	Vasculitis (SLE with CNS vasculitis)
20	24	F		-ESR 75, CRP 50.3 -CTA: severe stenosis of left VA, right CCA, left CCA, ICA and focal occlusion of left subclavian artery	Paresthesia at face and arm, left carotid bruit	Vasculitis (Takayasu arteritis)
21	86	F	HT, DM		Vertigo	Atherosclerosis
22	62	M	HT, DM, DLP		Left facial paresthesia	Atherosclerosis
23	47	F			Vertigo	Dissection
24	60	M	HT, smoking		Blurring vision	Atherosclerosis
25	66	F	HT, DM, DLP		Left hemiparesis	Atherosclerosis
26	63	M	HT, DM		Left hemiparesis	Atherosclerosis
27	81	F	HT, DLP		Ataxia	Atherosclerosis
28	84	F	HT, DM, DLP		Left facial paresthesia	Atherosclerosis
29	67	M	HT, DLP		Right hemiparesis	Atherosclerosis
30	40	F		-ESR 80 -CTA: moderate to severe stenosis at the left CCA, left ICA, bilateral subclavian and left vertebral arteries	Right hemiparesis, left carotid and subclavian bruit	Vasculitis (Takayasu arteritis)
31	72	M	HT, DM		Right hemiparesis	Atherosclerosis
32	42	M			Vertigo	Dissection
33	48	F			Vertigo	Dissection
34	77	M	HT, DM, DLP		Ataxia	Atherosclerosis
35	66	F		-ESR 87 -CSF: lymphocytic pleocytosis, elevated protein, normal glucose level	Left hemiparesis and dysarthria	Vasculitis (primary CNS vasculitis)
36	51	M		-ESR 62, CRP 5.79 -Positive anti-HIV -CSF: lymphocytic pleocytosis, elevated protein, low glucose level	Right hemiparesis and aphasia	Vasculitis (HIV with CNS vasculitis)
37	67	M	HT, DM, DLP		Left hemiparesis and dysarthria	Atherosclerosis
38	82	F	HT, DLP		Dysarthria	Atherosclerosis
39	53	F			Severe headache	Dissection
40	58	M	HT, DM, DLP		Right hemiparesis and dysarthria	Atherosclerosis
41	86	F	HT, DLP		Vertigo	Atherosclerosis
42	51	M	HT		Vertigo	Atherosclerosis
43	82	M			Ataxia	Dissection
44	69	F	DM, DLP		Ataxia	Atherosclerosis
45	53	M	DLP, smoking		Left hemiparesis	Atherosclerosis
46	72	M	HT, DM		Right hemiparesis	Atherosclerosis
47	59	M	HT, DLP		Left hemiparesis	Atherosclerosis
48	82	M	HT, DLP		Cognitive decline	Atherosclerosis
49	61	M	DLP		Right hemiparesis	Dissection
50	44	F			Right hemiparesis	Dissection
51	70	F	HT, DLP		Alteration of consciousness	Atherosclerosis
52	39	F			Left facial and arm paresthesia	Dissection
53	38	F		-ESR 90 -CSF: lymphocytic pleocytosis, elevated protein, elevated glucose level	Right arm weakness with slow speech	Vasculitis (primary CNS vasculitis)
54	15	F		-ESR 82 -Positive ANA, positive anti-DNA -CSF: lymphocytic pleocytosis, elevated protein, low glucose level	Drowsiness	Vasculitis (SLE with CNS vasculitis)
55	59	M	HT, smoking		Right arm weakness	Atherosclerosis
56	54	F		-ESR 70, CRP 10.49 -High serum IgG4 -Biopsy lymph node: IgG4-positive plasma cell -CSF: lymphocytic pleocytosis, elevated protein, normal glucose level	Progressive headache and seizure	Vasculitis (IgG4-associated CNS vasculitis)
57	64	M			Headache	Dissection
58	36	F			Right arm weakness	Dissection
59	25	M			Facial and trunk paresthesia	Dissection

*HT* hypertension, *DM* diabetes mellitus, *DLP* dyslipidemia, *CAD* coronary artery disease, *ESR* erythrocyte sedimentation rate, *CRP* C-reactive protein, *CSF* cerebrospinal fluid analysis, *ANA* antinuclear antibodies, *Anti-DNA* anti-DNA antibodies.

### Stenotic location and degree of stenosis

Interobserver agreement for stenotic location was excellent (*κ* = 0.91). In the atherosclerosis group, 26 (78.8%) patients showed diffuse stenosis, while seven (21.2%) patients showed focal stenosis. In the vasculitis group, the majority of patients showed diffuse stenosis (*n* = 10, 76.9%). For patients with dissection, all patients had focal stenotic involvement (*n* = 13, 100.0%), which were predominantly located in the internal carotid artery and the vertebral artery. There were significant differences in the stenotic location between atherosclerosis and dissection patients and between vasculitis and dissection patients (*P* < 0.001 for both). By contrast, there were no differences in the stenotic location between atherosclerosis and vasculitis patients (*P* = 1.00). The stenotic locations are shown in Table 2. The degree of arterial stenosis is shown in Table 3. Intraobserver agreement of the measured degree of arterial stenosis was excellent (*κ* = 0.98).

**Table 2:** Location of arterial stenosis in patients with atherosclerosis, vasculitis, and dissection

	Atherosclerosis (*n* = 33)	Vasculitis (*n* = 13)	Dissection (*n* = 13)	*P*-value			
				Ath vs Vas vs Dis	Ath vs Vas	Ath vs Dis	Vas vs Dis
**Diffuse**	26 (78.8%)	10 (76.9%)	0	< 0.001*	1.00	< 0.001*	< 0.001*
**Focal**	7 (21.2%)^a^	3 (23.1%)^b^	13 (100.0%)^c^				
**- CCA**	2/17 (11.8%)	0/9	0/14 (0.0%)				
**- ICA**^**+**^	6/17 (35.2%)	3/9 (33.3%)	8/14 (57.1%)				
**- ACA**	1/17 (5.9%)	3/9 (33.3%)	0/14				
**- MCA**	5/17 (29.4%)	3/9 (33.3%)	0/14				
**- PCA**	1/17 (5.9%)	0/9	0/14				
**- VA**^**+**^	2/17 (11.8%)	0/9	6/14 (42.9%)				
**Location of most stenotic point**				< 0.05*			
**- CCA**	5/33 (15.2%)	3/13 (23.1%)	0/13				
**- ICA**^**+**^	14/33 (42.4%)	4/13 (30.8%)	7/13 (53.8%)				
**- ACA**	0/33	2/13 (15.4%)	0/13				
**- MCA**	5/33 (15.2%)	3/13 (23.1%)	0/13				
**- PCA**	1/33 (3.0%)	0/13	0/13				
**- VA**^**+**^	8/33 (24.2%)	1/13 (7.7%)	6/13 (46.2%)				

^a^Seven cases with 17 lesions of focal involvement.

^b^Three cases with nine lesions of focal involvement.

^c^Thirteen cases with 14 lesions of focal involvement.

*Ath* atherosclerosis, *Vas* vasculitis, *Dis* dissection.

+ ICA, VA includes extracranial and/or intracranial segments.

*CCA* common carotid artery, *ICA* internal carotid artery, *ACA* anterior cerebral artery, *MCA* middle cerebral artery, *PCA* posterior cerebral artery, *VA* vertebral artery.

**Table 3:** Degree of arterial stenosis according to NASCET/WASID criteria

% of luminal narrowing	Atherosclerosis (*n* = 33)	Vasculitis (*n* = 13)	Dissection (*n* = 13)	*P*-value
Mild (0–49%)	5 (15.2%)	3 (23.1%)	2 (15.4%)	0.945
Moderate (50–70%)	8 (24.2%)	3 (23.1%)	2 (15.4%)	
Severe (70–99%)	8 (24.2%)	4 (30.8%)	3 (23.1%)	
Occlusion (100%)	12 (36.4%)	3 (23.1%)	6 (46.2%)	

### Vessel wall pattern

**Fig. 3:**
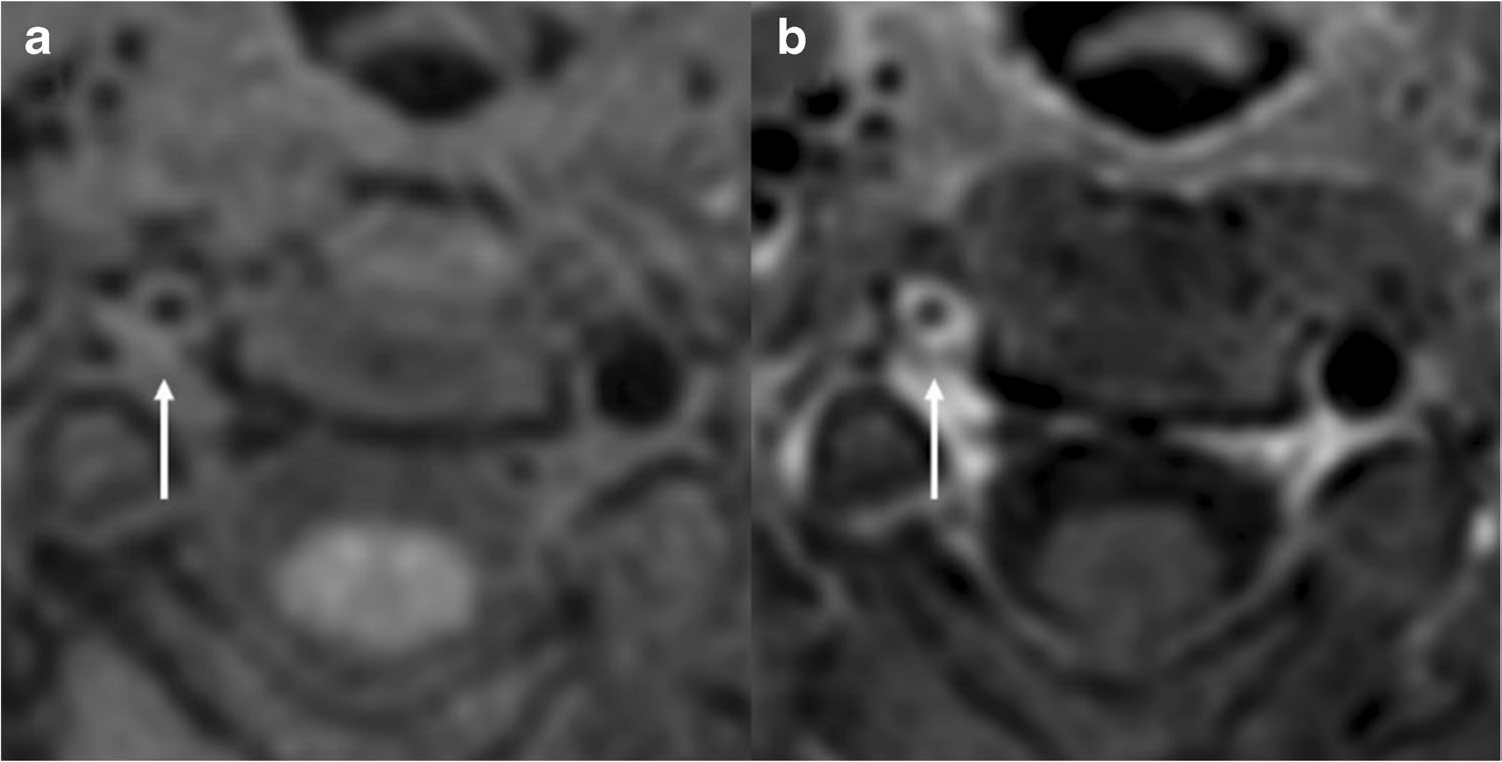
A 17-year-old woman with Takayasu arteritis. Pre-contrast (a) and CE-3D T1W VISTA (b) revealed concentric wall thickening with diffuse enhancement at the right VA (arrow)

**Fig. 4:**
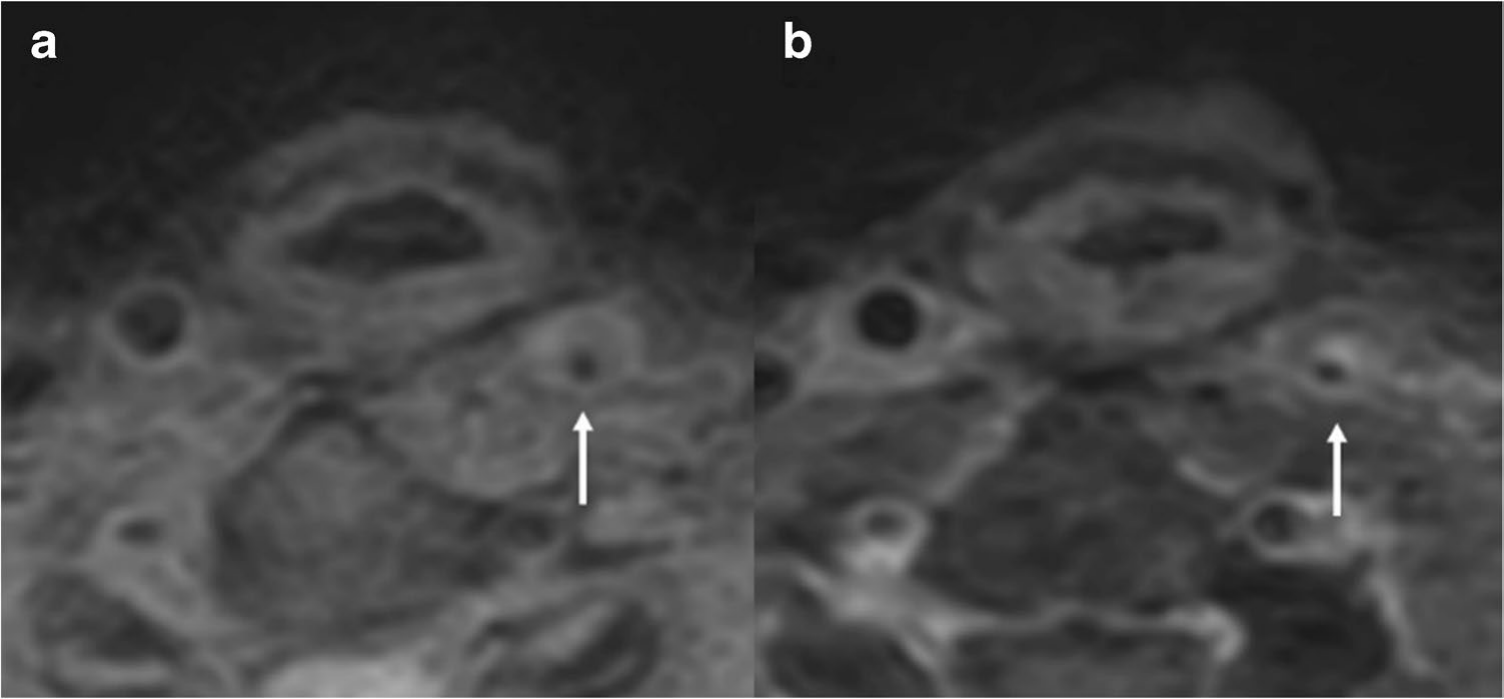
A 58-year-old man had an atherosclerotic plaque. Pre-contrast (a) and CE-3D T1W VISTA (b) demonstrated eccentric wall thickening with focal enhancement at the left cervical ICA (arrow)

The characteristic findings on the vessel wall images in each group are shown in Table 4. The pattern of involvement of the 11 vasculitis patients (84.6%) involved concentric luminal narrowing (Fig. 3). By contrast, only one of 33 atherosclerosis patients (3.0%) and one of 12 dissection patients (7.7%) showed concentric narrowing (*P* < 0.001). For wall enhancement, there was a significant difference between atherosclerosis and vasculitis patients (*P* < 0.05), with vasculitis patients mainly showing diffuse wall enhancement (*n* = 11, 84.6%) and atherosclerosis patients showing varied enhancement patterns (focal wall enhancement in 14 patients [42.4%], diffuse enhancement in 14 patients [42.4%], and no wall enhancement in five patients [15.2%]) (Fig. 4).

**Table 4:** Characteristics of vessel wall images in patients with atherosclerosis, vasculitis, and dissection

	Atherosclerosis (*n* = 33)	Vasculitis (*n* = 13)	Dissection (*n* = 13)	*P*-value			
				Ath vs Vas vs Dis	Ath vs Vas	Ath vs Dis	Vas vs Dis
**Pattern of involvement**				< 0.001*	< 0.001*	1.000	< 0.001*
**- Concentric**	1 (3.0%)	11 (84.6%)	1 (7.7%)				
**- Eccentric**	32 (97.0%)	2 (15.4%)	12 (92.3%)				
**Wall enhancement**				0.062	< 0.05*	0.421	0.073
**- Focal**	14 (42.4%)	1 (7.7%)	6 (46.2%)				
**- Heterogeneous**	0	0	0				
**- Diffuse**	14 (42.4%)	11 (84.6%)	7 (53.8%)				
**- Absent**	5 (15.2%)	1 (7.7%)	0 (0.0%)				
**Specific pattern**				< 0.001*	0.08	< 0.001*	< 0.001*
- IMH + flap*	0	0	3(23.1%)				
**- Intimal flap**	0	0	7 (53.8%)				
**- IPH**	6 (18.2%)	0	0				
**- Intraluminal clot**	0	1 (7.7%)	0				

*Note that no cases were found with an intramural hematoma (IMH) only. *Ath* atherosclerosis, *Vas* vasculitis, *Dis* dissection, *IPH* intraplaque hemorrhage.

**Fig. 5:**
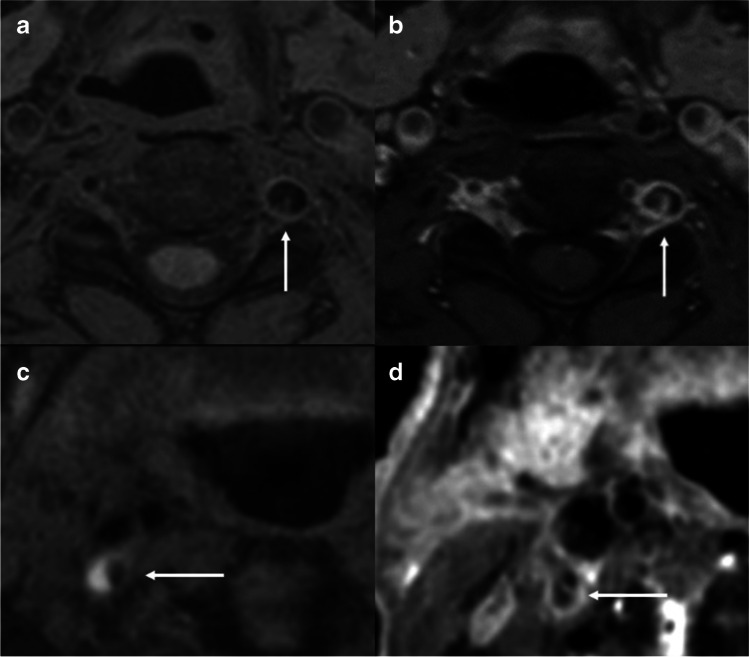
Pre-contrast (a) and CE-3D T1W VISTA (b) of a 43-year-old woman with spontaneous left VA dissection showed an enhanced curvilinear line of an intimal flap (arrow). Pre-contrast (c) and CE-3D T1W VISTA (d) of a 67-year-old man with traumatic dissection showed a hyperintense T1 signal indicating an intramural hematoma within the vessel wall (arrow, c) and the intimal flap (arrow, d)

An intraluminal flap was observed in 10 patients with dissection (76.9%). Of these patients, three had an intramural hematoma (23.1%) (Fig. 5).

Interobserver agreements were excellent for the pattern of involvement (*κ* = 0.89), with substantial agreement for the T1 signal (*κ* = 0.79), wall enhancement (*κ* = 0.72), and the specific pattern (*κ* = 0.74).

## Discussion

The main finding of the present study was that the majority of vasculitis patients exhibited concentric wall thickening (84.6%). These findings are similar to previous reports demonstrating a concentric wall thickening pattern in 67–100% of vasculitis patients [7–9, 11, 21]. The concentric thickened vessel wall in vasculitis is considered to be caused by intramural infiltration of inflammatory cells [16, 22, 23]. For atherosclerosis patients, many studies have reported eccentric wall thickening in 90–92% of cases [7, 8, 11, 24]. Furthermore, in a small case series evaluation of vertebrobasilar artery dissection using high-resolution VWI, Arai et al. [13] found an eccentric luminal narrowing in all five cases (100%). These studies agree with our findings of a wall involvement pattern, with a predominantly eccentric wall thickening in atherosclerosis (97.0%) and dissection (92.3%) patients. The eccentric wall thickening in atherosclerosis patients was also reported to be related to atherosclerotic plaques by histopathologic testing [10]. Arterial dissection caused by a tear of the intimal layer will create an intramural hematoma, resulting in an eccentric arterial wall appearance with the signal characteristics of blood [25].

We found no differences in wall enhancement between the three diseases. Nevertheless, subgroup analysis showed significant differences between atherosclerosis and vasculitis patients. For example, vasculitis patients tended to have diffuse wall enhancement (84.6%), while atherosclerosis patients demonstrated varied enhancement patterns. These findings are consistent with two studies by Mossa-Basha et al. [8, 11] that showed diffuse enhancement in vasculitis patients (86.5% and 100%), as well as focal (9.8% and 14.9%), diffuse (43.9% and 59.5%), heterogeneous (46.2% and 25.7%), and absent (5.2 and 15%) enhancement in atherosclerosis patients. In this study, we found no heterogeneous wall enhancement in atherosclerosis, vasculitis, and dissection. This finding was different from a previous study by Mossa-Basha et al. [8, 11] which could be due to the different method in visual inspection. In vasculitis, intramural inflammatory cell infiltration can cause mural integrity damage, resulting in contrast uptake [16, 22, 23]. However, the exact pathophysiological mechanisms of contrast uptake in atherosclerotic plaques remains unclear. Nevertheless, there is some evidence for inflammatory processes and neovascularization, which preferentially occur in the fibrous cap and adventitia or as an enhancement of the vasa vasorum [26, 27]. The presence of plaque enhancement also depends on plaque activity, with one meta-analysis reporting a robust association between plaque enhancement and recent ischemic events [28].

Another important finding of our study was the significant difference in the specific pattern between atherosclerosis, vasculitis, and dissection patients. For example, 23.1% of arterial dissection patients had an intramural hematoma, and 76.9% had an intimal flap (53.8% with an intimal flap only, 23.1% combined with an intramural hematoma). Wang et al. [12] also reported intimal flaps in 32 patients (42%) and intramural hematomas in 46 patients (61%). Furthermore, Han et al. [14] reported that an intimal flap was the most frequent finding, present in 91.4% of patients, while 54.3% of patients had an intramural hematoma. The rate of intramural hematoma was lower in our study. As previously described, the intramural hematoma signal is dependent on the stage of blood, which evolves with time [28, 29]. The T1-hyperintense signal of the intramural hematoma relates to the T1 shortening effect of methemoglobin, which is observed in the subacute stage of blood. The mean duration from visiting time to VWI time of our dissection patients was 50.15 ± 33.45 days (min–max: 3–92 days; five cases at < 4 weeks, one case at 4–6 weeks, one case at 6–8 weeks, and six cases at > 8 weeks). In the three patients who received intramural hematoma MRI, imaging was performed on the 7th, 19th, and 21st day after presentation. One case, who was taken for MRI at 3 days from symptom onset, showed only a minimal T1-hyperintensity, which was not interpreted as an intramural hematoma. Another patient who received MRI on the 26th day after presentation did not show any T1-hyperintensity at the stenotic point. Thus, the correlation between the signal characteristics of the intramural hematoma and the exact duration requires further clarification.

We also found a significant difference in the location of involvement between the three disease groups. The majority of stenotic lesions in vasculitis patients were found in a diffuse fashion, similar to atherosclerosis; this may be explained by the systemic nature of these two diseases, which result in widespread vessel involvement. By contrast, dissection occurred spontaneously or by trauma and was typically focal. Every dissection patient in our study showed focal vessel involvement—the internal carotid artery and the vertebral artery were the predominant locations. Our study also provided evidence for the utility of high-resolution VWI in differentiating between atherosclerosis, vasculitis, and dissection by using the vessel wall pattern. For example, there were significant differences in the pattern of involvement and in the specific pattern between the three groups, a significant difference in the wall enhancement pattern between atherosclerosis and vasculitis patients in subgroup analysis, and a significant difference in the location of involvement.

There are some limitations of this study. First, this was a retrospective study with a small sample size (there were only 13 cases of dissection and vasculitis). Second, we did not routinely perform T2 VWI in every patient. Further studies are required to confirm the T2 signal differences between these diseases.

## Conclusion

Assessment of the pattern of involvement, wall enhancement, and the specific patterns using vessel wall MRI is useful for differentiating between atherosclerosis, vasculitis, and dissection. Concentric wall thickening with diffuse enhancement in vasculitis patients, eccentric wall thickening with a diffuse location in atherosclerosis patients, and an intimal flap in arterial dissection patients should be used to distinguish these vasculopathies.

## Data Availability

The datasets generated during and/or analysed during the current study are available from the corresponding author on reasonable request.

## Abbreviations

MR: Magnetic resonance
VWI: Vessel wall imaging
